# Ethanol Molecule Engineering Toward Stabilized 1T-MoS_2_ with Extraordinary Sodium Storage Performance

**DOI:** 10.3390/molecules30183801

**Published:** 2025-09-18

**Authors:** Xue’er Bi, Xuelian Wang, Xiaobo Shen, Haijun Yu, Xian Zhang, Jin Bai

**Affiliations:** 1 School of Electronic Engineering, Huainan Normal University, Huainan 232038, China; 17364431095@163.com (X.B.); shenxb@hnnu.edu.cn (X.S.); haijun20030@163.com (H.Y.); zhangxian035@163.com (X.Z.); 2 School of Materials Science and Engineering, Anhui University, Hefei 230601, China; 3 Key Laboratory of Materials Physics, Institute of Solid State Physics, The Hefei Institutes of Physical Science (HFIPS), Chinese Academy of Sciences, Hefei 230031, China; 4 Anhui Province Key Laboratory of Low-Temperature Co-Fired Materials, Huainan Normal University, Huainan 232038, China

**Keywords:** 1T-MoS_2_, ethanol molecule engineering, elaborated nanosheets structure, MoS_2_ nanoclusters, sodium-ion batteries

## Abstract

Phase molybdenum disulfide (1T-MoS_2_) holds significant promise as an anode material for sodium-ion batteries (SIBs) due to its metallic conductivity and expanded interlayer distance. However, the practical application of 1T-MoS_2_ is hindered by its inherent thermodynamic metastability, which poses substantial challenges for the synthesis of high-purity, long-term stable 1T phase MoS_2_. Herein, a synergetic ethanol molecule intercalation and electron injection engineering is adopted to induce the formation and stabilization of 1T-MoS_2_ (E-1T MoS_2_). The obtained E-1T MoS_2_ consists of regularly arranged sphere-like ultrasmall few-layered 1T-MoS_2_ nanosheets with expanded interlayer spacing. The high intrinsic conductivity and enlarged interlayer spacing are greatly favorable for rapid Na^+^ or e^−^ transport. The elaborated nanosheets structure can effectively relieve volume variation during Na^+^ intercalating/deintercalating processes, shorten transport path of Na^+^, and enhance diffusion kinetics. Furthermore, a novel sodium reaction mechanism involving the formation of MoS_2_ nanoclusters during cycling is revealed to produce the higher surface pseudocapacitive contribution to Na^+^ storage capacity, accelerating Na^+^ reaction kinetics, as confirmed by the kinetics analysis and ex-situ structural characterizations. Consequently, the E-1T MoS_2_ electrode exhibits an excellent sodium storage performance. This work provides an important reference for synthesis and reaction mechanism analysis of metastable metal sulfides for advanced SIBs.

## 1. Introduction

Sodium-ion batteries (SIBs) are increasingly recognized as a promising technology for large-scale energy storage applications, primarily driven by the abundance of sodium resources and their inherent cost advantages [1,2,3,4]. However, the practical application of SIBs faces significant challenges. The larger ionic radius (~1.06 Å, 1 Å = 10^−10^ m) and higher molar mass of Na^+^ compared to Li^+^ seriously induces substantial electrode volume changes, including expansion and potential pulverization, resulting in sluggish diffusion kinetics [5,6]. Consequently, identifying suitable electrode materials, particularly for the key anode materials, is crucial. While graphite serves as the commercial anode in lithium-ion batteries, it exhibits extremely low capacity for sodium storage, rendering it unsuitable for SIBs [7,8]. Similarly, commercially available hard carbon anodes, despite their usefulness, struggle to meet the escalating demands for high energy density SIBs due to their limited capacities [9,10]. This context underscores the imperative to develop novel high-performance anode materials tailored for advanced SIBs.

Molybdenum disulfide (MoS_2_) has emerged as a promising anode candidate for SIBs, largely attributed to its distinctive layered architecture. The relatively large interlayer spacing (~0.62 nm), combined by weak van der Waals (vdW) forces, facilitates rapid sodium ion (de)intercalation and underpins a high theoretical capacity of 670 mAh g^−1^ [11,12,13,14]. MoS_2_ primarily exists in two distinct phases, 2H and 1T, dictated by variations in Mo-S atomic coordination and stacking sequences [15]. The thermodynamically stable 2H-MoS_2_ phase adopts a trigonal prismatic coordination, exhibiting semiconducting behavior characterized by a bandgap of ~1.9 eV [16]. However, its application as a SIB anode is still hampered by inherent limitations: sluggish Na^+^ diffusion kinetics, poor reversibility in Mo-S bonding, and significant volume expansion during cycling. These factors collectively lead to rapid capacity degradation [17,18]. While extensive strategies, such as nanostructuring [19,20] and carbon compositing [21,22] have been employed to enhance sodium storage performance of 2H-MoS_2_, its intrinsic properties pose fundamental challenges, resulting in sodium storage capabilities that fall short of practical requirements. In contrast, the 1T-MoS_2_ features an octahedral coordination structure and exists in a thermodynamically metastable state. Crucially, it exhibits metallic conductivity, surpassing that of the 2H phase by 10^5^ to 10^7^ times [23,24]. This exceptional electrical conductivity, coupled with a larger interlayer spacing (~1 nm), makes 1T-MoS_2_ particularly advantageous for facilitating rapid Na^+^ and electron transport, positioning it as an exceptionally suitable anode material for SIBs [25,26]. Nevertheless, the inherent metastability of the 1T phase presents a significant synthesis challenge, as it tends to degradation toward 2H-MoS_2_ under ambient conditions, thereby severely limiting its practical utility.

To achieve stable 1T-MoS_2_ synthesis for enhanced sodium storage performance, diverse strategies have been explored. These include alkali metal intercalation [27], organic solvent-assisted exfoliation [28], and heteroatom doping [29]. Yet despite the effectiveness of these proposed strategies in enhancing the sodium storage performance of MoS_2_, intrinsic challenges still exist in the resulting 1T-MoS_2_ anodes. These typically involve complex and time-consuming synthesis procedures, or manifest as lower 1T phase purity, long-term stability, and production yields. Such limitations hinder their large-scale application [30,31]. Consequently, developing an efficient and scalable approach to obtain high-purity and highly stable 1T-MoS_2_ for SIBs anodes remains a significant challenge. Recently, the electron injection strategy could be promising as an effective method to obtain high-purity and highly stable 1T-MoS_2_ [32]. For instance, Zhang et al. [33] developed an electron-modulated and phosphate radical-stabilized approach to produce stable 1T-rich MoS_2_, accelerating Na^+^ insertion/extraction kinetics and yielding ultrahigh rate capability. Similarly, He et al. [34] utilized facile electron injection engineering to fabricate a TiO_2_-1T MoS_2_ nanoflower composite, which reduced the Na^+^ diffusion barrier and suppressed sulfur dissolution, leading to outstanding rate performance and cycling stability. Sun et al. [35] synthesized a defective, 1T-rich MoS_2_/m-C nanoflower composite, which facilitated rapid charge transport, resulting in high sodium storage capacity, superior rate capability, and excellent long-term cycling stability. The above-listed studies employed some smaller organic molecules or cations/anions, such as ethylene glycol, ascorbic acid, or Ni^2+^/PO_4_^3−^, that were introduced into the hydrothermal synthesis of MoS_2_, inserting the interlayer of MoS_2_ and inducing the formation of 1T-MoS_2_. However, the organic molecules derived carbon or inactive cations/anions could influence full delivery of specific capacity of the obtained 1T-MoS_2_ electrode. How to achieve the formation of the 1T-MoS_2_ through the induction of an external medium without affecting its full performance potential might be of great significance. Thus, the ethanol molecule could be appropriately considered due to the fact that the smaller ethanol molecule not only easily entered the interlayer, but also did not remain in the interlayer to derive the carbon layer due to its extremely low boiling point, which is promising for obtaining high-purity 1T-MoS_2_ to realize full capacity release. Furthermore, ethanol is cleaner and more environmentally friendly compared to other small alcohol molecules (e.g., methanol, isopropanol), which could be more suitable for large-scale sample synthesis and potential battery application.

Herein, we developed an ethanol molecule assisted solvothermal method and realized a stable E-1T MoS_2_ via a synergetic ethanol molecule intercalation and electron injection engineering. The intercalation of the ethanol molecule and its -OH group as an electron donor facilitates the formation and stabilization of 1T-MoS_2_. The obtained E-1T MoS_2_ exhibits the sphere-like nanoflower structure composed of the regularly arranged few-layered ultrasmall 1T-MoS_2_ nanosheets. Benefiting from the synergetic effect of the high electrical conductivity, expanded interlayer spacing and regularly arranged nanosheets structure, the E-1T MoS_2_ electrode displays an excellent sodium storage performance with the high reversible capacity of 459.7 mAh g^−1^ after 200 cycles at 1 A g^−1^ and remarkable rate capability of 312.2 mAh g^−1^ at 10 A g^−1^.

## 2. Results and Discussion

**Figure 1:**
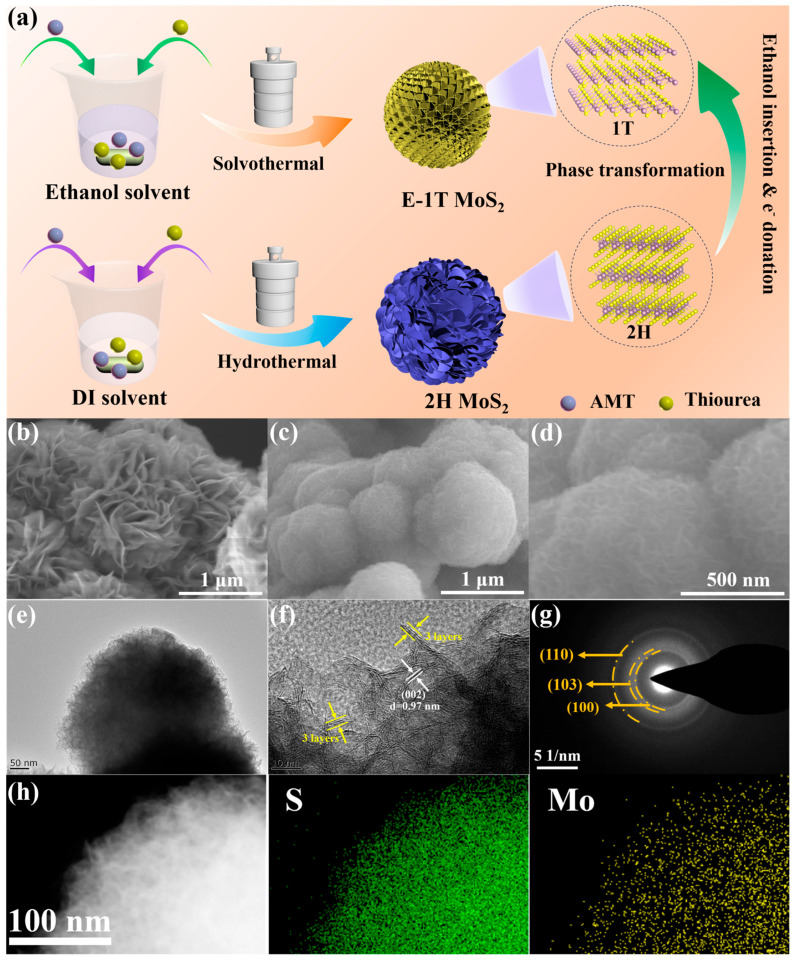
(**a**) Schematic illustration of the synthesis of E-1T MoS_2_ and 2H MoS_2_ samples. SEM images of (**b**) 2H MoS_2_ sample, and (**c**,**d**) E-1T MoS_2_ sample at the different magnifications. (**e**) TEM image, (**f**) HRTEM image, (**g**) SAED pattern, and (**h**) EDS mapping images of E-1T MoS_2_ sample.

The synthesis processes of E-1T MoS_2_ and 2H MoS_2_ samples are schematically illustrated in Figure 1a. First, the ammonium molybdate (AMT) and thiourea were mixed into the deionized (DI) water via magnetic stirring, and then underwent a typical hydrothermal process to obtain a pristine 2H MoS_2_ sample with an irregular nanoflower-like structure. When the DI water solvent was replaced by the ethanol solvent, the similar solvothermal process was performed to achieve an extraordinary E-1T MoS_2_ sample with a regular sphere-like interlaced nanosheets structure. The transformation mechanism from 2H to 1T phase could be attributed to the ethanol molecules intercalating into the interlayer of MoS_2_ grain in the solvothermal process, and its -OH functional group serving as an electron donor to induce the formation of 1T phase with expanded interlayer spacing, which could be universal for the general alcohol-based small molecules [32,35]. Moreover, the low-boiling point ethanol molecule did not remain in the interlayer, which is beneficial to full delivery of high sodium storage capacity discussed below. Meanwhile, the rich -OH functional groups brought by the ethanol molecules could also act as nucleation sites to confine the growth of MoS_2_ nanosheets and prevent their irregular stack, forming the regular nanosphere-like structure assembled by ultrasmall nanosheetsFigure 1b and Figure S1 clearly show the different magnification SEM images of the 2H MoS_2_ sample exhibiting the nanoflower-like morphology with a diameter of about 1–2 μm, composed of many irregularly aggregated nanosheets with length sizes of around 400–500 nm. As shown in Figure 1c,d, the E-1T MoS_2_ sample presents the smaller micron-sized nanosphere-like shape due to the smaller nanosheet unit with a lateral size of about 50–100 nm. Figure 1e also exhibits the TEM image of E-1T MoS_2_ sample, further demonstrating a sphere-like morphology consisting of many regularly interwoven nanosheets with lateral sizes of around 50 nm and thicknesses of about 2–3 nm. The HRTEM image of E-1T MoS_2_ sample (Figure 1f) displays the few-layer nanosheet character, with about three layers, accompanied by a larger interlayer spacing of around 0.97 nm. As shown in the in-plane HRTEM image of the E-1T MoS_2_ sample (Figure S2), there exists hexagonal and trigonal lattice areas representing 2H and 1T phase structure characteristics, respectively, suggesting the co-existence of 1T and 2H phases in the E-1T MoS_2_ sample. Meanwhile, the 2H MoS_2_ sample exhibits the thicker multi-layer nanosheet structure with smaller interlayer spacing of about 0.67 nm (Figure S3), proving the intercalation and electron transfer of the ethanol molecule induced the formation of 1T phase with the larger interlayer spacing, which is beneficial to Na^+^ transport kinetics as discussed below. Figure 1g and Figure S4 show the selected area electron diffraction (SAED) patterns of the E-1T MoS_2_ and 2H MoS_2_ samples, respectively, all exhibiting the concentric ring characteristic of polycrystalline structures. Compared to the obvious concentric rings in 2H MoS_2_ sample (Figure S4), the SAED pattern of E-1T MoS_2_ became weaker and its (002) diffraction ring almost disappeared, indicating the significantly reduced nanosheet size and few-layer nanosheet structure, which is also consistent with the XRD and TEM results. The EDS mapping images display the homogeneous distribution of Mo and S elements, indicating the reliable MoS_2_ structure.

**Figure 2:**
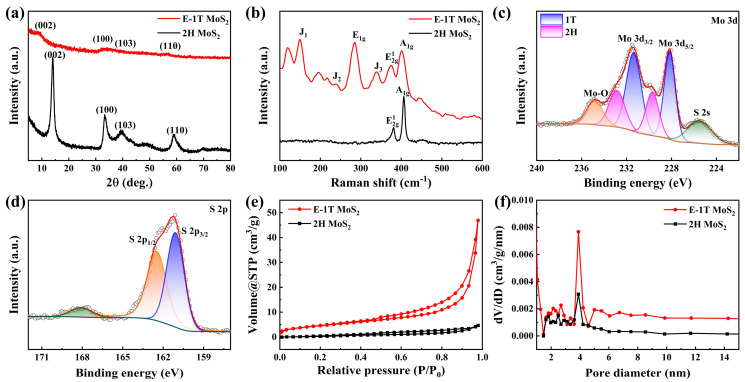
(**a**) XRD patterns and (**b**) Raman spectra of E-1T MoS_2_ and 2H MoS_2_ samples. High-resolution XPS spectra of (**c**) Mo 3d and (**d**) S2p of E-1T MoS_2_ sample. (**e**) Isothermal N_2_ adsorption/desorption curves and (**f**) pore size distributions of E-1T MoS_2_ and 2H MoS_2_ samples.

Figure 2a shows the XRD patterns of 2H MoS_2_ and E-1T MoS_2_ samples. The pristine 2H MoS_2_ sample exhibits the four obvious diffraction peaks, which can be well indexed to the standard JCPDS Card No. 75-1539, indicating a pure hexagonal phase structure [36]. Compared to 2H MoS_2_ sample, the XRD diffraction peaks of the E-1T MoS_2_ sample became significantly weak, and a new (002) peak at about 8.8° appeared, suggesting the formation of the ultrasmall few-layer 1T-MoS_2_ nanosheets structure with low crystallinity. For the obvious left shift of main (002) peak, we also calculated the corresponding interlayer spacing (d) based on Bragg’s law: 2dsinθ = λ (for detailed calculation, see Note S2), increasing it from 0.63 to 1 nm. Thus, we speculated that the expanded interlayer spacing and reduced nanosheet size could be attribution to the intercalation and confined growth of the ethanol molecules, respectively, which is aligned with the TEM and HRTEM results. To further understand the interesting formation mechanism of 1T-MoS_2_, the XRD patterns comparison of E-1T MoS_2_ samples with the different ethanol concentrations were also provided (Figure S5). The results indicate that the full ethanol solvent environment could induce the ultrasmall few-layer 1T-MoS_2_ nanosheets structure. Furthermore, for 1T-MoS_2_, 1T phase stability over time is critical. Thus, we have provided the XRD patterns comparison of E-1T MoS_2_ sample at fresh synthesis state and after nine months, as shown in Figure S6. The results indicate there are no obvious changes for the XRD patterns of the sample from the two time periods, indicating the excellent 1T phase ability of the obtained E-1T MoS_2_ sample. The Raman spectra of both samples are shown in Figure 2b. For the 2H MoS_2_ sample, the two obvious vibration peaks are located at about 380.5 and 406.2 cm^−1^, which can be attributed to $E_{2g}^{1}$ and A_1g_ modes of 2H phase MoS_2_ [37]. Meanwhile, for the E-1T MoS_2_ sample, the new evident characteristic peaks of the main E_1g_ mode (~284.9 cm^−1^), and three additional J_1_ (~148.5 cm^−1^), J_2_ (~238.1 cm^−1^), and J_3_ (~338.1 cm^−1^) modes can be observed, which correspond to vibration modes of 1T-MoS_2_ [38]. Furthermore, an XPS test was conducted to explore the compositions and phase structures. Figure S7 displays the survey XPS spectrum of the E-1T MoS_2_ sample, which is mainly composed of Mo and S elements. Figure 2c and Figure S8 display the high-resolution Mo 3d spectra of E-1T MoS_2_ and 2H MoS_2_ samples, respectively. Compared to Mo 3d_3/2_ (232.9 eV) and Mo 3d_5/2_ (229.7 eV) characteristic peaks of 2H MoS_2_ sample (Figure S8), the Mo3d spectrum of the E-1T MoS_2_ sample can be deconvoluted into the four main characteristic peaks at 231.4 and 228.2 eV (indexed to 1T-MoS_2_), and 232.9 and 229.7 eV (assigned to 2H-MoS_2_) [39]. The peak positions of Mo 3d in E-1T MoS_2_ sample shifted toward the lower binding energies, indicating enhanced Mo charge density due to the introduction of the ethanol molecules [40]. Moreover, based on the fitting results of Mo 3d spectrum in the E-1T MoS_2_ sample, the 1T phase proportion can be calculated to be as high as 70%. Furthermore, we have provided the comparison of the efficiency and phase purity of E-1T MoS_2_ in previous studies [33,34], as shown in Table S1. The results indicate that the synthesis efficiency of our E-1T MoS_2_ sample is much higher just via a one-step solvothermal process, compared to the two previous studies (two-step or multi-step synthesis process). Although 1T phase purity is only 70% in the obtained E-1T MoS_2_ sample, higher than that of this reported work [33] and lower than that of another work [34], it still demonstrated the highest synthesis efficiency and excellent sodium storage performance discussed below, indicating its high cost-effectiveness for SIBs anode toward practical application. Besides, the remarkable phase stability after nine months and high yield via solvothermal method further emphasize its practical value. The additional peaks at about 225.5 and 234.8 eV correspond to S 2s and Mo–O bonding, respectively. Compared to the 2H MoS_2_ sample, the formation of the obvious Mo–O bonding suggests that -OH functional group in the ethanol molecule bridged MoS_2_ counterpart and inserted ethanol molecule, realizing the electron transfer to induce the formation of 1T phase [41]. In addition, the S 2p spectrum of the E-1T MoS_2_ sample can be fitted into the two obvious characteristic peaks at 162.5 and 161.1 eV, as shown in Figure 2d, which are attributed to S 2p_1/2_ and S 2p_3/2_ of S^2−^, respectively. Figure 2e and f show the isothermal N_2_ adsorption/desorption curves and corresponding pore size distributions of 2H MoS_2_ and E-1T MoS_2_ samples, respectively. As shown in Figure 2e, the E-1T MoS_2_ sample displays a more evident H3-type hysteresis loop compared to the 2H MoS_2_ sample, indicating a richer mesoporous structure. Furthermore, the Bruauer–Emmett–Teller (BET) specific areas of both samples can be calculated to be about 31.3 and 7.0 m^2^/g for the E-1T MoS_2_ and 2H MoS_2_ samples, respectively. The increased specific area for E-1T MoS_2_ could be related to the regularly arranged nanosheets structure and corresponding reduced nanosheets size as demonstrated in SEM and TEM analyses. Moreover, the E-1T MoS_2_ sample has more abundant pore diameter distributions and larger pore volume than the 2H MoS_2_ sample based the Barret–Joyner–Halenda (BJH) method (Figure 2f), which will promote the Na^+^ diffusion ability as discussed below.

**Figure 3:**
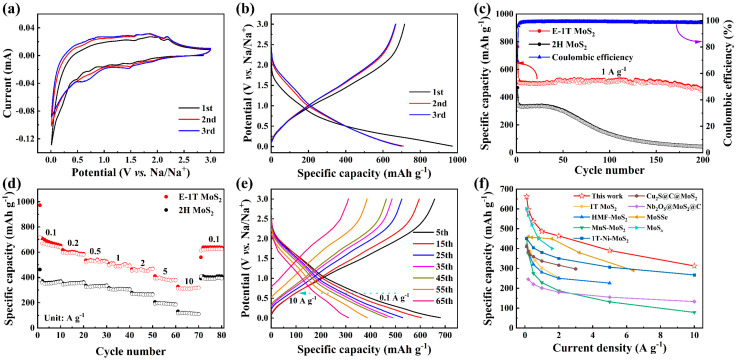
Initial three cycles of (**a**) CV curves at 0.1 mV s^−1^ and (**b**) GCD profiles at 0.1 A g^−1^ of E-1T MoS_2_ electrode. (**c**) Cycling performance of E-1T MoS_2_ and 2H MoS_2_ electrodes at 1 A g^−1^. (**d**) Rate performance of E-1T MoS_2_ and 2H MoS_2_ electrodes. (**e**) GCD profiles of E-1T MoS_2_ electrode at the various current densities. (**f**) Rate capability comparison of E-1T MoS_2_ electrode with the other reported MoS_2_-based electrodes for SIBs.

The sodium storage performance of the E-1T MoS_2_ electrode was evaluated via assembling a 2032 coin-type half-cell with Na foil as a counter electrode. Figure 3a and Figure S8 show the first three cycles CV curves of E-1T MoS_2_ and 2H MoS_2_ electrodes at 0.1 mV s^−1^, respectively. Compared to the CV curves of the 2H MoS_2_ electrode with the typical reversible conversion reaction process from MoS_2_ to Mo and Na_2_S (Figure S9), the redox peaks of the CV curves in the E-1T MoS_2_ electrode became less obvious, indicating the enhanced pseudocapacitive behavior, discussed below, which could be originated from the regularly arranged ultrasmall nanosheets structure with the larger specific area. Meanwhile, in the first cathodic scanning, the weak reduction peak at 0.21 V is attributed to the conversion reaction toward metallic Mo and Na_2_S, accompanied with the formation of the solid electrolyte interphase (SEI) film [42]. In the subsequent cathodic scanning, the reduction peaks shifted upward to about 1.51 and 0.65 V, which correspond to the insertion of Na^+^ and follow conversion reaction processes, respectively. In the corresponding anodic scanning, the oxidation peaks of about 1.84 and 2.18 V can be attributed to the gradual Na^+^ desertion and reversible conversion reaction processes. Moreover, the subsequent CV curves overlapped well, indicating the excellent electrochemical reaction reversibility. Figure 3b and Figure S10 display the GCD profiles for first three cycles of the E-1T MoS_2_ and 2H MoS_2_ electrodes at 0.1 A g^−1^. The E-1T MoS_2_ electrode delivered the high initial discharge and charge specific capacities of 972.0 and 716.3 mAh g^−1^, much higher than those of the 2H MoS_2_ electrode (462.0 and 381.2 mAh g^−1^). Moreover, the subsequent GCD curves almost coincide, further suggesting the remarkable reaction reversibility of E-1T MoS_2_ electrode, which agrees well with the above CV analysis. Furthermore, it can be seen that there only appear some weak inclined plateaus in the GCD curves in the E-1T MoS_2_ electrode, which correspond to the extremely weak redox peaks in the CV curves, further indicating the strong pseudocapacitive behavior. Figure 3c displays a comparison of the cycling performance of the E-1T MoS_2_ and 2H MoS_2_ electrodes at 1 A g^−1^ (red arrow corresponds to the left coordinate axis, and blue arrow corresponds to the right coordinate axis). The E-1T MoS_2_ electrode exhibits an outstanding reversible capacity of 459.7 mAh g^−1^ after 200 cycles at 1 A g^−1^, accompanied with a high capacity retention of 86.8%. Meanwhile, the specific capacities of the E-1T MoS_2_ electrode exhibited obvious fluctuations, and demonstrated an evident capacity decay after 200 cycles, which decreased to 375.2 mAh g^−1^ after 250 cycles, with a lower capacity retention of 70.8% (Figure S11). Furthermore, to emphasize potential practical value of E-1T MoS_2_ electrode, its cycling performance demonstrated a higher loading mass of about 4 mg cm^−2^, also shown in Figure S12. The results indicate that the E-1T MoS_2_ electrode with the higher area loading can still deliver a high specific capacity of about 400 mAh g^−1^ at 5 A g^−1^ after 100 cycles. However, its cycling performance is still unsatisfactory and obvious capacity decay is apparent after 120 cycles, which is consistent with that of the above E-1T MoS_2_ electrode with the low area loading. The capacity decay could be attributed to the conversion reaction mechanism between Mo/Na_2_S and MoS_2_ accompanied by the larger volume variation, resulting in a cycling performance that is worthy of further improvement. However, the pristine 2H MoS_2_ electrode displays more inferior cycling performance, which maintains a stable capacity of 329.5 mAh g^−1^ in the former 40 cycles and then exhibits a significant capacity fading in the subsequent cycles. It retains an unsatisfactory capacity of 43.6 mAh g^−1^ after 200 cycles at 1 A g^−1^, with an extremely low cycling retention of about 13.2%. Furthermore, the comparison of the coulombic efficiencies (CE) of both electrodes during long-term cycling were also provided in Figure S13 (for CE calculation, see Note S3). The results indicate the E-1T MoS_2_ electrode exhibits relatively more stable CE values than the pristine 2H MoS_2_ electrode, further indicating the superior electrochemical reversibility. The comparison of the rate capability for E-1T MoS_2_ and 2H MoS_2_ electrodes is displayed in Figure 3d. The E-1T MoS_2_ electrode shows excellent rate performance with the reversible capacities of 662.0, 596.0, 537.1, 487.1, 463.5, and 390.1 mAh g^−1^ at the respective current densities of 0.1, 0.2, 0.5, 1, 2, and 5 A g^−1^. Even at a high current density of 10 A g^−1^, a prominent capacity of 312.2 mAh g^−1^ can still be obtained. Nevertheless, the 2H MoS_2_ electrode shows a poor rate capability of 119.7 mAh g^−1^ at 10 A g^−1^. To intuitively demonstrate the enhanced rate capability for E-1T MoS_2_ electrode, its GCD curves at the various current densities were plotted in Figure 3e. It can be seen that the GCD curves overlap well with a smaller voltage polarization from 0.1 to 10 A g^−1^, accompanied with a high rate capacity retention of about 47.2%, magnifying a rapid Na^+^ diffusion kinetics, which is consistent with the rate performance analysis. Impressively, the rate capability of the E-1T MoS_2_ electrode outperforms those of many previously reported MoS_2_-based anodes for SIBs, as shown in Figure 3f. To emphasize the practicality of E-1T MoS_2_ anode for SIBs, the corresponding full cell was also assembled via E-1T MoS_2_ as anode and Na_3_V_2_(PO_4_)_3_ as cathode. Figure S14a shows the GCD curves for first three cycles of the E-1T MoS_2_ ‖ NVP full cell within 0.5–3.5 V at 0.1 A g^−1^, displaying normal charge/discharge behavior within voltage range and outputting the sloping charge and discharge plateaus at about 2.3 and 1.7 V, respectively, in the subsequent cycles. The cycling performance of the full cell at 0.1 A g^−1^ is displayed in Figure S14b. It displayed a high initial charge capacity beyond 400 mAh g^−1^, which could be attributed to the formation of SEI film on the anode side contributing to the higher irreversible capacity. It also delivered an initial discharge capacity of about 223.6 mAh g^−1^, which slightly decreased to 105.8 mAh g^−1^ after 100 cycles, accompanied by a capacity retention of about 47.3%. The lower specific capacity of the full cell compared to that of the half cell could be closely related to the operation voltage range of the anode in the full cell. Based on the charge and discharge output voltages of about 2.3 and 1.7 V, respectively, it can be determined that only the capacity within 0–1.5 V or even a narrower voltage range can be contributed to the full cell, leading to the obtained specific capacities lower than 400 mAh g^−1^. Furthermore, the present specific capacity and retention remain limited, yet they clearly demonstrate the feasibility of applying E-1T MoS_2_ in full-cell devices and highlight directions for further optimization, including capacity matching between anode and cathode, voltage window, and anode performance.

**Figure 4:**
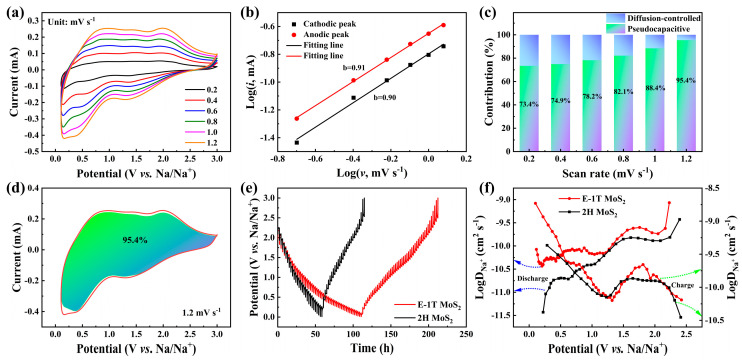
(**a**) CV curves of E-1T MoS_2_ electrode at the different scan rates. (**b**) The calculated b values for E-1T MoS_2_ electrode via Log(*i*)-Log(*v*) relationship. (**c**) Pseudocapacitive contribution ratios of E-1T MoS_2_ electrode at the various scan rates. (**d**) The fitting of pseudocapacitive contribution ratio at 1.2 mV s^−1^. (**e**) GITT curves and (**f**) Na^+^ diffusion coefficients during the discharging and charging processes of E-1T MoS_2_ and 2H MoS_2_ electrodes.

To elucidate the enhanced Na^+^ reaction kinetics of the E-1T MoS_2_ electrode, CV measurements were conducted at various scan rates ranging from 0.2 to 1.2 mV s^−1^ (Figure 4a). Despite increasing scan rates, the CV profiles retain similar shapes. However, they exhibit progressively larger peak currents and minimal peak shift, indicating reduced polarization. Furthermore, the relationship between the peak current (*i*) and scan rate (*v*) follows a power-law behavior described by Equation (1): [43]

*i* = a*v*^b^ (1)

where a represents dimensionless coefficient, b = 1.0 signifies pseudocapacitive charge storage, b = 0.5 indicates diffusion-limited behavior, and 0.5 < b < 1.0 denotes a mixed charge storage mechanism. By analyzing the anodic and cathodic peaks across multiple scan rates, the linear relationship between log(*i*) and log(*v*) was plotted and fitted as presented in Figure 4b. The calculated b values are about 0.90 and 0.91 for the cathodic and anodic peaks, respectively, thus confirming a surface pseudocapacitance-dominated charge storage mechanism in the E-1T MoS_2_ electrode. To further quantify the pseudocapacitive contribution, the current response (*i*) can be deconvoluted as a function of scan rate (*v*) using Equation (2) [44]

*i* = k_1_*v* + k_2_*v*^1/2^ (2)

where k_1_*v* corresponds to the pseudocapacitive contribution, while k_2_*v*^1/2^ represents the diffusion-controlled contribution. Figure 4c illustrates the calculated pseudocapacitive contributions for the E-1T MoS_2_ electrode across varying scan rates. Notably, this contribution increases progressively with increasing scan rates, reaching ratios of 73.4%, 74.9%, 78.2%, 82.1%, 88.4%, and 95.4% at 0.2, 0.4, 0.6, 0.8, 1.0, and 1.2 mV s^−1^, respectively. Specifically, at 1.2 mV s^−1^, a representative CV profile was fitted to intuitively demonstrate this behavior (Figure 4d), revealing a dominant surface pseudocapacitance contribution of 95.4% (for detailed fitting process, see Note S4). Such a high surface contribution even at a lower scan rate of about 1 mV s^−1^ could be a common phenomenon in the modified electrode materials for batteries [29,33,35]. Furthermore, the CV curves within ultrahigh scanning rates of 10–100 mV s^−1^ (Figure S15) no longer represent charge storage behavior of the electrode for batteries, but rather the charge storage behavior similar to that of in supercapacitors, accompanied by significant variation of shapes and currents of CV curves compared to those of 0.2–1.2 mV s^−1^, leading to the failed simultaneous pseudocapacitive fitting with those of within 0.2–1.2 mV s^−1^, which is originated from the failed linear fitting for obtaining k_1_ values via the fitting process shown in Note S4, thus failing to obtain continuous pseudocapacitive contribution variations from 0.2 to 100 mV s^−1^. While the pseudocapacitive contributions at 10–100 mV s^−1^ can still be obtained, the fitting process is only carried out within this scanning rate range, obtaining a pseudocapacitive contribution that is no more than 70% at most, as also demonstrated by the previous studies on most MoS_2_-based electrodes materials for supercapacitors [45], which seems to be contradictory with the pseudocapacitive fitting results obtained at 0.2–1.2 mV s^−1^. It could be that, at ultrahigh scan rates, the ion diffusion process cannot be completed within such an extremely short timescale, leading to surface-limited charge accumulation that resembles supercapacitor-type behavior rather than true battery-type pseudocapacitance. The above results further indicate that overhigh scanning rates could be unsuitable for studying pseudocapacitive contribution of the present E-1T MoS_2_ electrode for SIBs. Meanwhile, the manifestation of pseudocapacitance could be different between battery-type and supercapacitor-type systems even on the same MoS_2_-based electrode materials, deserving further deep clarification in the future. Inspiringly, this high surface pseudocapacitance contribution of E-1T MoS_2_ for SIBs significantly enhances rapid Na^+^ transport kinetics, particularly at high current densities, underpinning the superior rate capability demonstrated earlier. The origin of this high surface pseudocapacitance primarily stems from the regularly arranged sphere-like ultrasmall nanosheet structure and former nanoclusters during cycling, as discussed below, facilitating rapid surface sodium adsorption/desorption processes, which is fully consistent with the CV analysis. To further understand the elevated Na^+^ diffusion kinetics of the E-1T MoS_2_ electrode with the outstanding rate capability, galvanostatic intermittent titration technique (GITT) measurements of E-1T MoS_2_ and 2H MoS_2_ electrodes were conducted, and are displayed in Figure 4e. The E-1T MoS_2_ electrode exhibits the longer discharge/charge time and smaller voltage drop compared to the 2H MoS_2_ electrode. Furthermore, the diffusion coefficient (*D*_Na_^+^) can be calculated based on Equation (3): [46]

(3) $$D=\frac{4}{\pi \tau }(\frac{m_{B}V_{m}}{M_{B}S}{)}^{2}(\frac{\Delta E_{S}}{\Delta E_{\tau }}{)}^{2}$$

where *m_B_*, *V_m_*, *M_B_*, and *S* are mass, molar volume, molar mass and contact area of electrode material, respectively. *τ*, Δ*E_S_*, and Δ*E_τ_* are pulse current time, voltage variations of steady state, and pulse process, respectively. As shown in Figure 4f (blue arrow corresponds to the left coordinate axis, and green arrow corresponds to the right coordinate axis), the *D*_Na_^+^ values of E-1T MoS_2_ electrode during discharging and charging processes were calculated to be about 10^−9^~10^−10^ and 10^−8^~10^−10^ cm^2^ s^−1^, respectively, evidently higher than those of 2H MoS_2_ electrode (10^−10^~10^−11^ and 10^−9^~10^−11^ cm^2^ s^−1^), indicating the significantly enhanced Na^+^ diffusion kinetics of E-1T MoS_2_ electrode, which could be attributed to the synergetic effect between the metallic 1T phase and regularly arranged ultrasmall nanosheet structure.

**Figure 5:**
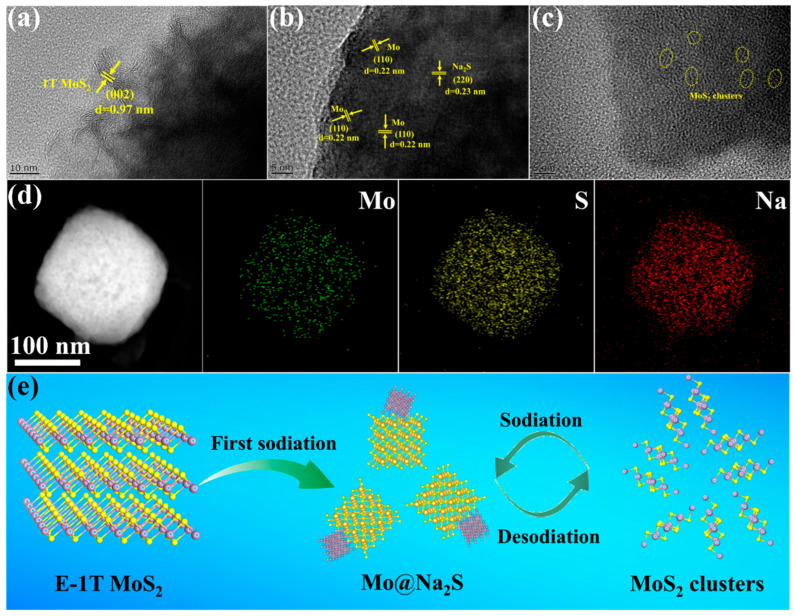
Ex-situ TEM images at (**a**) OCV, (**b**) fully sodiation and (**c**) fully desodiation states for E-1T MoS_2_ electrode. (**d**) ex-situ EDS mapping images of E-1T MoS_2_ electrode at fully sodiation state. (**e**) The schematic diagram of sodium reaction mechanism of E-1T MoS_2_ electrode.

To further reveal the sodium storage mechanism of the optimal E-1T MoS_2_ electrode during the sodiation/desodiation processes, ex-situ XRD measurements at the different discharging/charging states were performed to explore its phase structure evolution, as shown in Figure S16. At the open circuit voltage (OCV) state, The E-1T MoS_2_ electrode still displays the similar weak XRD pattern with its powder sample. When discharged from OCV to 0.01 V, several obvious peaks representing Na_2_S species appeared, indicating the phase transformation from MoS_2_ to Mo and Na_2_S during the sodiation process. When charged to 3 V, the XRD peaks of Na_2_S disappeared, while there appeared no obvious peaks of MoS_2_, indicating the amorphous character of the reconstructed MoS_2_ structure, and possible conversion reaction from Mo and Na_2_S to MoS_2_ nanoclusters, which will be further verified below. To further explore the phase structure evolution in E-1T MoS_2_ electrode in-depth, ex-situ TEM tests were also conducted, as shown in Figure 5a–c. At OCV state, the HRTEM image of E-1T MoS_2_ electrode still exhibits the few-layer ultrasmall 1T-MoS_2_ nanosheets structure with the larger interlayer spacing of about 0.97 nm, which is consistent with its powder sample (Figure 5a). At the fully discharged state of 0.01 V, the typical lattice fringes of (002) planes in the HRTEM image of E-1T MoS_2_ electrode disappeared, while two sets of new lattice fringes appeared, consisting of the smaller particle fringes of about 0.22 nm and larger continuous fringes of about 0.23 nm (Figure 5b), indicating the formed metallic Mo particles became embedded into the Na_2_S matrix. Moreover, the EDS-mapping images at the fully discharged states (Figure 5d) exhibit the uniform distribution of Mo, S, and Na elements, further suggesting the conversion reaction mechanism during the sodiation process. Moreover, we have provided the ex-situ TEM measurements of the different regions in the E-1T MoS_2_ electrode at the fully discharged state as shown in Figure S17. The results indicate that there exist obvious SEI film layers coated on the surface of the E-1T MoS_2_ electrode after the sodiation reaction process, which could be beneficial to the cycling stability of the electrode, further proving the above conversion reaction process accompanied by the formation of SEI film. Furthermore, at the fully charged state of 3 V, the ex-situ HREM image in Figure 5c displays there appeared many ultrasmall 1–2 layer nanosheets stripes with size of about 2 nm (yellow dotted circles) in the electrode, which are short-term ordered but long-term disordered. Thus, we vividly referred to it as “MoS_2_ nanoclusters”. The above results indicate the reversible transformation of Mo and Na_2_S species toward MoS_2_, as confirmed by the EDS-mapping images in Figure S18, which could contribute the more rapid pseudocapacitive behavior to sodium storage capacity as discussed above. The ex-situ EDS mapping images at the fully charged state exhibit the same distribution of Mo and S elements, while the emerged dispersive distribution of Na element could be attributed to the formed SEI film on the electrode, further suggesting the reversible conversion reaction process accompanied by SEI film formation. Moreover, the ex-situ SAED pattern at the fully de-sodiation state (Figure S19) presents a fuzzy central spot, further confirming the formed MoS_2_ nanoclusters with the amorphous character during the charging process. To intuitively reflect the sodium storage mechanism of the E-1T MoS_2_ electrode, a schematic illustration is presented in Figure 5e. During the first sodiation process, E-1T MoS_2_ converted to the metallic Mo and embedded into Na_2_S. The Mo and Na_2_S species performed the reversible conversion reaction toward the MoS_2_ nanoclusters during the subsequent sodiation/desodiation processes. Based on the above sodium storage mechanism, the excellent sodium storage performance of E-1T MoS_2_ electrode could be assigned to the following aspects: (1) the induced metallic 1T phase structure with the large interlayer spacing and high electrical conductivity is beneficial to the rapid transfer of Na^+^ or e^-^, promoting sodium reaction kinetics; (2) the regular sphere-like ultrasmall nanosheets structure provides more sodium storage active sites, shortens the diffusion path of Na^+^ and accommodates the larger volume expansion during the cycling process; (3) the formed MoS_2_ nanoclusters display the stronger surface pseudocapacitance behavior, leading to the enhanced Na^+^ reaction kinetics.

## 3. Materials and Methods

**Materials synthesis:** First, in a typical solvothermal procedure, the molybdenum and sulfur precursors of 0.3506 g ammonium molybdate tetrahydrate (NH_4_)_6_Mo_7_O_24_·4H_2_O) and 0.599 g thiourea were dissolved in 18.3 mL of ethanol under constant magnetic stirring for 1 h. The resulting homogeneous mixture was subsequently sealed within a Teflon-lined autoclave and subjected to a thermal treatment at 210 °C for 18 h within a conventional oven. Upon cooling to ambient temperature, the black precipitate was collected and thoroughly washed using deionized (DI) water and ethanol, applied alternately over three times. Final drying was conducted in a vacuum oven at 60 °C for 12 h, yielding the ethanol-induced 1T MoS_2_ nanosheets (designated E-1T MoS_2_). The chemical reagents and instruments in the materials synthesis were all from Sinopharm Group Chemical Reagent Co., Ltd., Shanghai, China, and Shanghai Jinghong Experimental Equipment Co., Ltd., Shanghai, China, respectively.

**Materials characterizations:** The crystallographic structure and phase purity of the synthesized samples were evaluated using powder X-ray diffraction (XRD, Cu Kα radiation, XpertProMPD, PANalytical B.V., Almelo, Netherlands), with diffraction patterns recorded over a 2θ range within 5–80°. Raman spectroscopy was performed on a spectrometer (T6400, Horiba Jobin Yvon, Shanghai, China) employing a 514.5 nm laser excitation source. Sample morphology and microstructural features were investigated utilizing field-emission scanning electron microscopy (FE-SEM, SU8020, Hitachi Ltd., Tokyo, Japan), transmission electron microscopy (TEM, Tecnai G2F20, JEOL Ltd., Tokyo, Japan), and corresponding high-resolution TEM (HRTEM) analyses. Elemental composition was determined via energy-dispersive X-ray spectroscopy (EDS) integrated with the TEM system. Surface chemical states were probed by X-ray photoelectron spectroscopy (XPS) using an instrument (Thermo Scientific ESCALAB 250, Shanghai, China) equipped with an Al Kα X-ray source. The specific surface area and pore size distribution were determined by nitrogen adsorption–desorption isotherms measured at 77 K using Brunauer–Emmett–Teller (BET) and Barrett–Joyner–Halenda (BJH) methods (ASAP2460, Micromeritics, Shanghai, China).

**Electrochemical measurements:** The sodium storage capabilities were assessed using CR2032 coin cells assembled within an argon-filled glove box (<0.1 ppm H_2_O/O_2_). Sodium metal foil served as the counter/reference electrode. For working electrode preparation, the active material, conductive carbon (Super P), and binder (sodium carboxymethyl cellulose, CMC) were homogeneously blended with an 8:1:1 mass ratio in DI water. The resulting slurry was then uniformly coated onto a copper foil current collector and subsequently vacuum-dried at 80 °C for 12 h. The loading mass of the working electrodes is about 1–4 mg/cm^2^, and the calculation of specific capacity is based on the loading mass of active material (for determining method, see Note S1). The coin-type cells employed glass microfiber filters as separators and utilized an electrolyte consisting of 1 M sodium perchlorate (NaClO_4_) dissolved in a binary solvent mixture of ethylene carbonate (EC) and propylene carbonate (PC) (1:1 of volume ratio), supplemented with 5 wt% fluoroethylene carbonate (FEC) additive. Galvanostatic charge-discharge (GCD) profiles, cycling stability, and rate capability assessments were conducted within a voltage window of 0.01 to 3.0 V (vs. Na^+^/Na) using a Neware battery testing system. Additionally, cyclic voltammetry (CV) measurements were acquired on a CHI 660E electrochemical workstation, scanning between 0.01 and 3.0 V at varying scan rates. The full cell performance was further evaluated using E-1T MoS_2_ as the anode material and self-made Na_3_V_2_(PO_4_)_3_ (NVP) as the cathode material. Meanwhile, the NVP cathode slurry was prepared by combining Na_3_V_2_(PO_4_)_3_ powder, Super P, and polyvinylidene fluoride (PVDF) binder in a weight ratio of 7:2:1, dissolved/dispersed in N-methyl-2-pyrrolidone (NMP) solvent. This slurry was uniformly coated onto aluminum current-collecting foil and then dried overnight to obtain the NVP cathode. The obtained NVP cathode demonstrated a working voltage of about 3.3–3.4 V. The GCD profiles and cycling performance of the full cell were tested within a voltage window of 0.5 to 3.5 V at the current density of 0.1 A g^−1^. The loading masses of the anode and cathode materials in the full cell configuration were set at about 1 and 4 mg cm^−2^, respectively, to ensure a negative-to-positive (N/P) capacity ratio maintained at about 1.1:1. The reported specific capacity of the full cell was calculated based on the loading mass of the anode material. The raw materials and instruments in the electrochemical measurements were all from Suzhou Duoduo Chemical Technology Co., Ltd., Suzhou, China, and Shanghai Chenhua Instrument Co., Ltd., Shanghai, China, respectively.

## 4. Conclusions

In summary, a synergetic ethanol molecule intercalation and electron injection engineering is employed to construct stable E-1T MoS_2_ just via a facile and effective ethanol molecule assisted solvothermal process. The ethanol molecule intercalated the interlayer of MoS_2_ and its -OH group performed electron donation to induce the formation and stabilization of 1T-MoS_2_. The morphological and structural characterizations display that the obtained E-1T MoS_2_ possesses the regularly arranged sphere-like ultrasmall few-layered 1T-MoS_2_ nanosheets structure with the enlarged interlayer spacing. The high intrinsic conductivity, expanded interlayer spacing, and tailored nanosheets structure of E-1T MoS_2_ synergistically promoted Na^+^ diffusion kinetics and enhanced structural stability during Na^+^ insertion/extraction processes. Moreover, the kinetics analysis, ex-situ XRD, and TEM measurements unraveled a novel sodium reaction mechanism involving formation of MoS_2_ nanoclusters, leading to a stronger surface pseudocapacitance behavior and accelerating Na^+^ reaction kinetics. As a result, the E-1T MoS_2_ electrode displays an excellent cycling stability of 459.7 mAh g^−1^ after 200 cycles at 1 A g^−1^ and outstanding rate capability of 312.2 mAh g^−1^ at 10 A g^−1^. This simple and effective ethanol molecule engineering could provide guidance for the design of 1T phase metal sulfides for high-performance SIBs.

## Data Availability

The original contributions presented in this study are included in the article/Supplementary Materials. Further inquiries can be directed to the corresponding authors.

## Supplementary Materials

The following supporting information can be downloaded at: https://www.mdpi.com/article/10.3390/molecules30183801/s1, Figure S1: High-magnification SEM image of 2H MoS_2_ sample; Figure S2: In-plane HRTEM image of E-1T MoS_2_ sample; Figure S3: HRTEM image of 2H MoS_2_ sample; Figure S4: SAED pattern of 2H MoS_2_ sample; Figure S5: XRD patterns comparison of E-1T MoS_2_ samples with the different ethanol concentrations; Figure S6: XRD patterns comparison of E-1T MoS_2_ sample at fresh synthesis state and after 9 months; Figure S7: Survey XPS spectrum of E-1T MoS_2_ sample; Figure S8: High-resolution Mo 3d spectrum of E-1T MoS_2_ sample; Figure S9: Initial three cycles CV curves of 2H MoS_2_ electrode; Figure S10: First three cycles GCD curves of 2H MoS_2_ electrode; Figure S11: Cycling performance of E-1T MoS_2_ electrode after the longer 250 cycles at 1 A g^−1^; Figure S12: Cycling performance of E-1T MoS_2_ electrode with high areal loading about 4 mg cm^−1^ at 1 A g^-1^; Figure S13: Coulombic efficiencies comparison of E-1T MoS_2_ and 2H MoS_2_ electrodes after 500 cycles at 1 A g^−1^; Figure S14: (a) First three cycles GCD curves and (b) cycling performance of the E-1T MoS_2_ ‖ NVP full cell at 0.1 A g^−1^; Figure S15: CV curves of E-1T MoS_2_ electrode within the range of 10–100 mV s^−1^; Figure S16: Ex-situ XRD patterns of E-1T MoS_2_ electrode at the different discharge/charge states; Figure S17: Ex-situ TEM images of the different regions in E-1T MoS_2_ electrode at the fully discharged state; Figure S18: Ex-situ EDS mapping images of E-1T MoS_2_ electrode at fully desodiation state; Figure S19: Ex-situ SAED pattern of E-1T MoS_2_ electrode at fully desodiation state; Table S1: Comparison of synthesis efficiency and 1T phase purity for E-1T MoS_2_ and previously reported 1T-MoS_2_ samples; Note S1: The calculation process of loading mass of electrode material; Note S2: The calculation process of d-spacings of the samples; Note S3: The calculation of coulombic efficiency; Note S4: The fitting method of pseudocapacitive contributions.
