# Supplementary material for: Ethanol Molecule Engineering Toward Stabilized 1T-MoS2 with Extraordinary Sodium Storage Performance
Source: Molecules. 2025 Sep 18;30(18):3801. doi: 10.3390/molecules30183801 (PMC12472969; doi:10.3390/molecules30183801)
Supplement: Supplementary file 1 [file molecules-30-03801-s001.zip › molecules-3848360-supplementary.pdf]

Ethanol Molecule Engineering Toward Stabilized 1T-MoS_2 with Extraordinary Sodium Storage Performance

Xue'er Bi [1], Xuelian Wang [1,2,3,4,*], Xiaobo Shen [1], Haijun Yu [1], Xian Zhang [1,4] and Jin Bai [3,*]

1 School of Electronic Engineering, Huainan Normal University, Huainan 232038, China; 17364431095@163.com (X.B.); shenxb@hnnu.edu.cn (X.S.); haijun20030@163.com (H.Y.); zhangxian035@163.com (X.Z.)
2 School of Materials Science and Engineering, Anhui University, Hefei 230601, China
3 Key Laboratory of Materials Physics, Institute of Solid State Physics, The Hefei Institutes of Physical Science (HFIPS), Chinese Academy of Sciences, Hefei 230031, China
4 Anhui Province Key Laboratory of Low-Temperature Co-Fired Materials, Huainan Normal University, Huainan 232038, China
* Correspondence: wangxuelian@mail.ustc.edu.cn (X.W.), jbai@issp.ac.cn (J.B.)

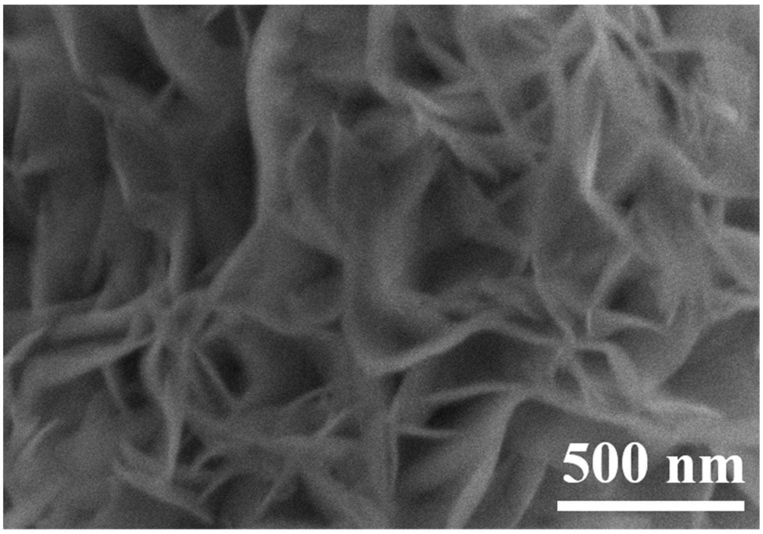

Figure S1. High-magnification SEM image of 2H MoS_2 sample.

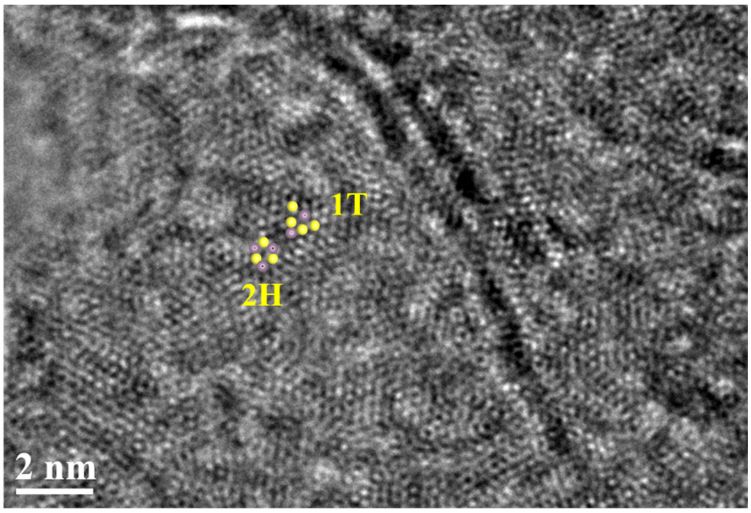

Figure S2. In-plane HRTEM image of E-1T MoS_2 sample.

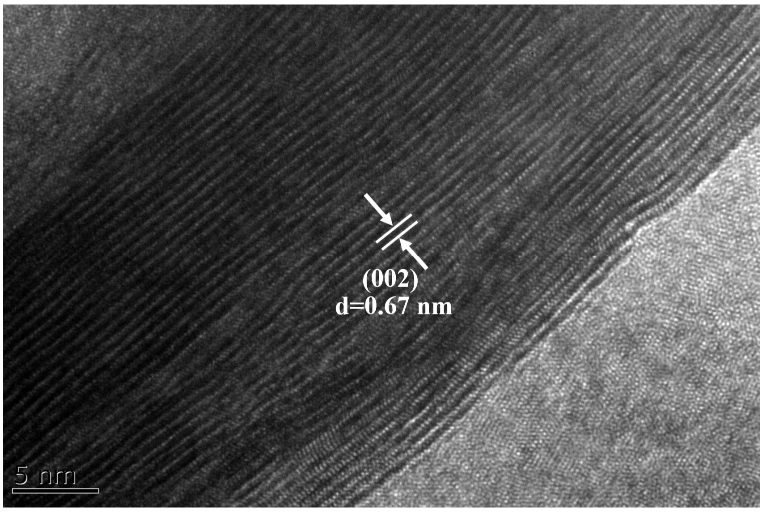

Figure S3. HRTEM image of 2H MoS_2 sample.

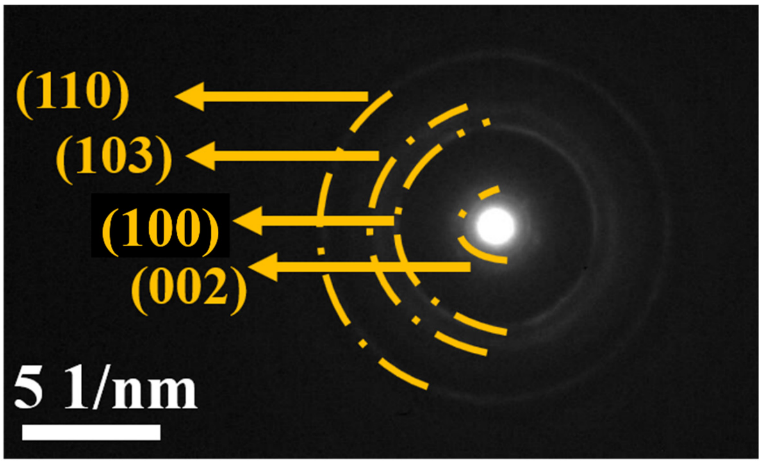

Figure S4. SAED pattern of 2H MoS_2 sample.

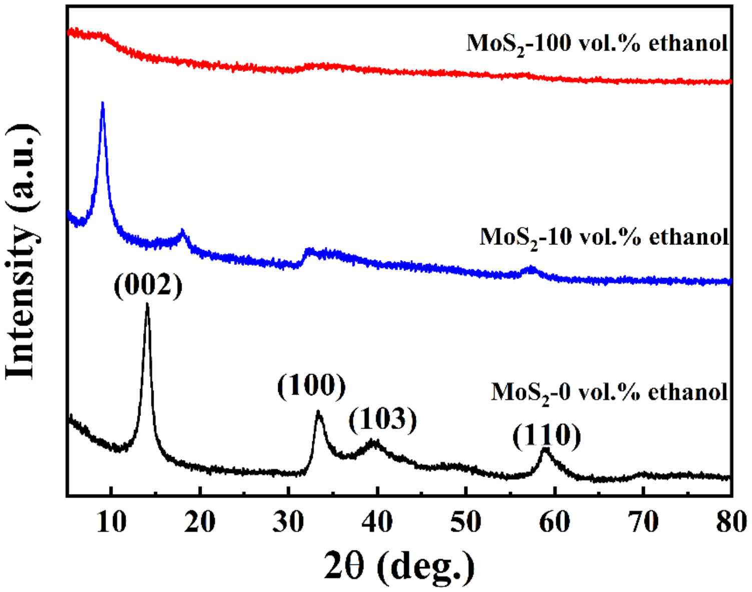

Figure S5. XRD patterns comparison of E-1T MoS_2 samples with the different ethanol concentrations.

We provided the XRD patterns comparison of E-1T MoS_2 samples with the different

ethanol concentrations (0, 10 and 100 vol.%). The result indicates that when the solvent is pure deionized water, that is, 0 vol.% ethanol, MoS_2 exhibits the XRD pattern of 2H phase MoS_2 structure. And when the ethanol concentration was increased to 10 vol.%, the XRD pattern of 1T phase MoS_2 structure emerges with the stronger diffraction peaks, especially for the main (002) peak, indicating the larger multi-layer nanosheets structure. While increased to 100 vol.%, the diffraction peaks of 1T phase MoS_2 still exist, but become extremely weak, suggesting the full ethanol environment significantly confined the growth of nanosheets and inducing the formation of the ultrasmall few-layered 1T phase nanosheets structure. Besides, the obtained E-1T MoS_2 sample with 100 vol.% ethanol concentration own the optimal sodium storage performance.

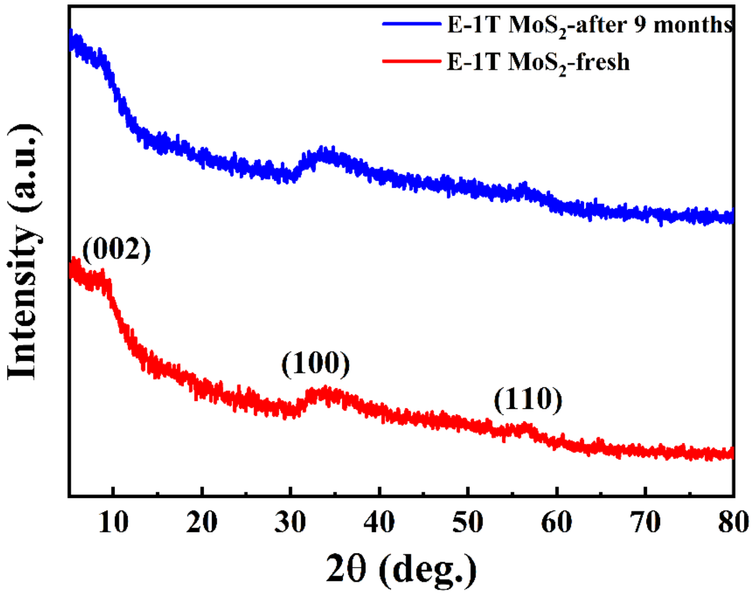

Figure S6. XRD patterns comparison of E-1T MoS_2 sample at fresh synthesis state and after 9 months.

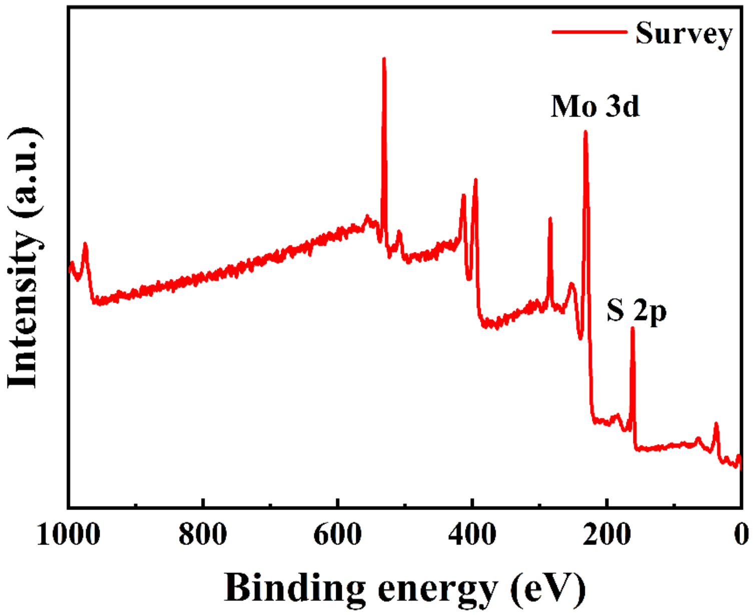

Figure S7. Survey XPS spectrum of E-1T MoS_2 sample.

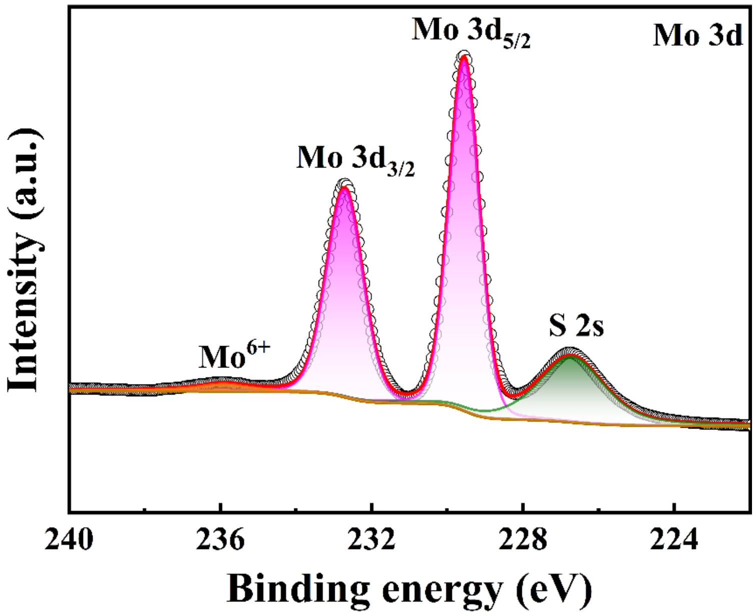

Figure S8. High-resolution Mo 3d spectrum of E-1T MoS_2 sample.

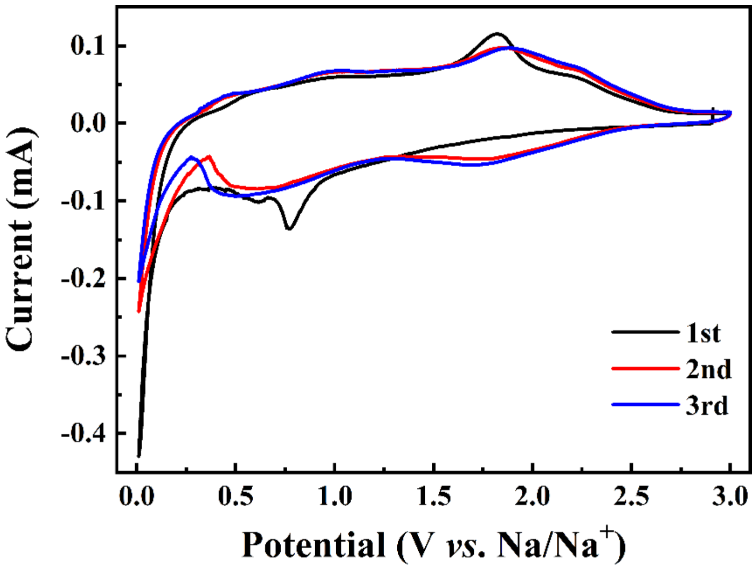

Figure S9. Initial three cycles CV curves of 2H MoS_2 electrode.

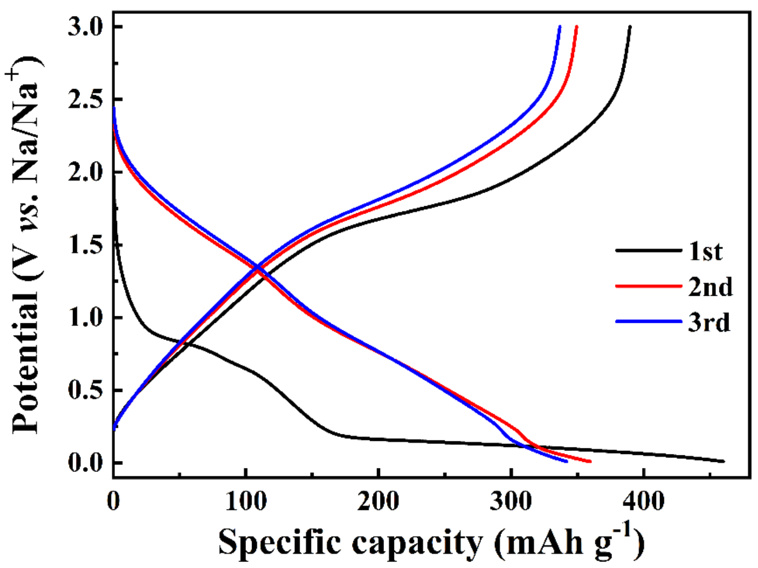

Figure S10. First three cycles GCD curves of 2H MoS_2 electrode.

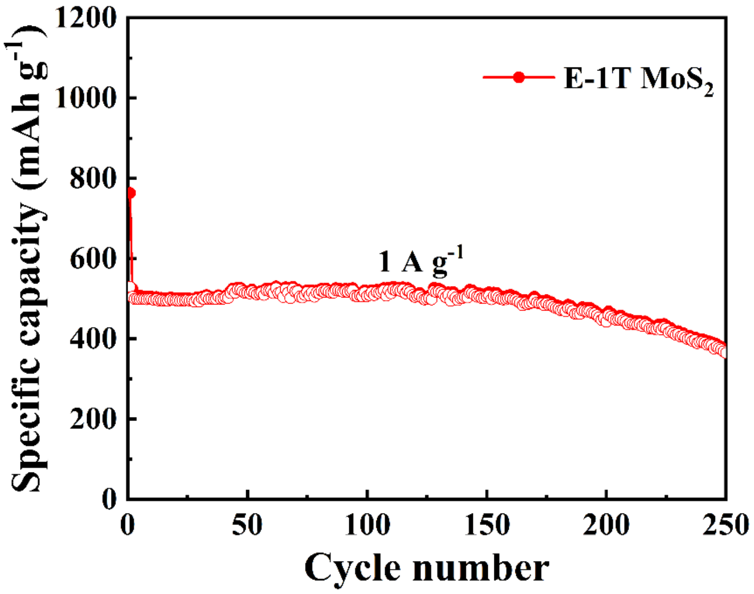

Figure S11. Cycling performance of E-1T MoS_2 electrode after the longer 250 cycles at 1 A g^{-1}.

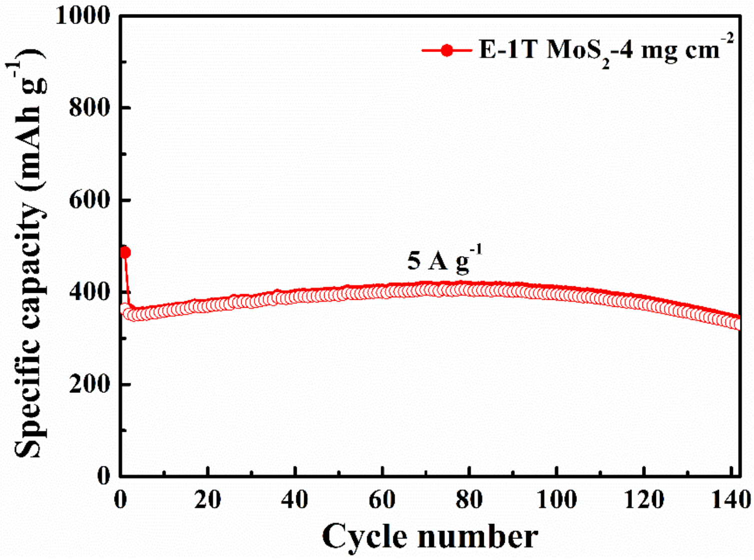

Figure S12. Cycling performance of E-1T MoS_2 electrode with high areal loading about 4 mg cm^{-1} at 1 A g^{-1}.

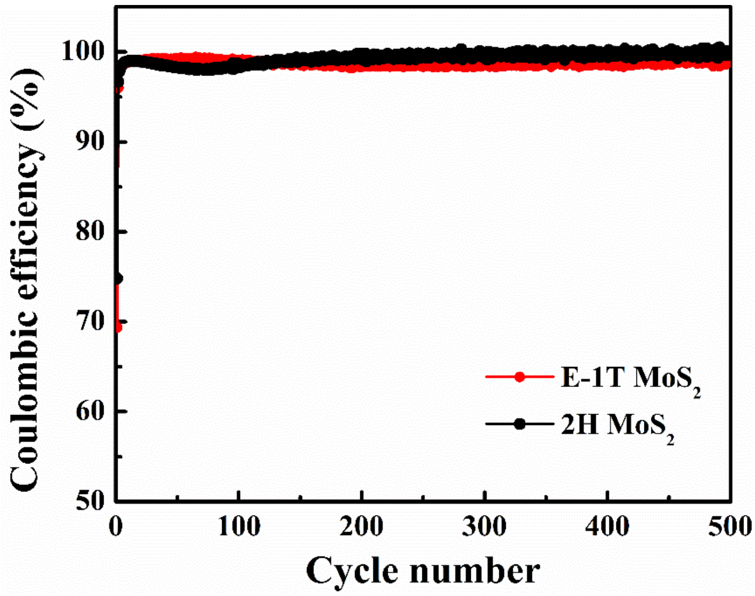

Figure S13. Coulombic efficiencies comparison of E-1T MoS_2 and 2H MoS_2 electrodes after 500 cycles at 1 A g^{-1}.

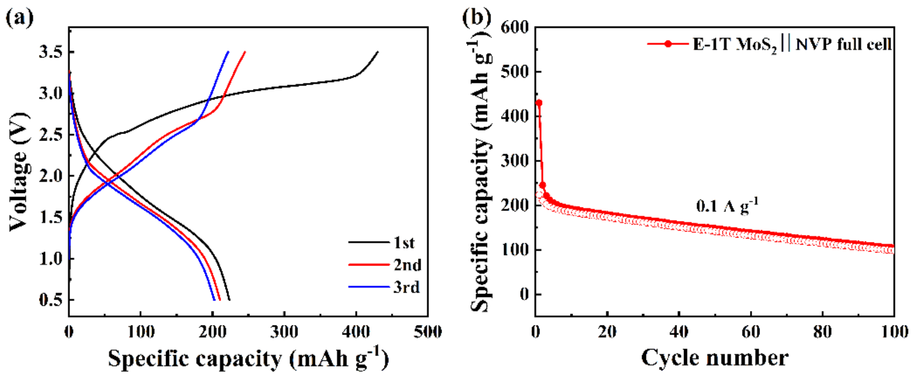

Figure S14. (a) First three cycles GCD curves and (b) cycling performance of the E-1T MoS_2 ‖ NVP full cell at 0.1 A g^{-1}.

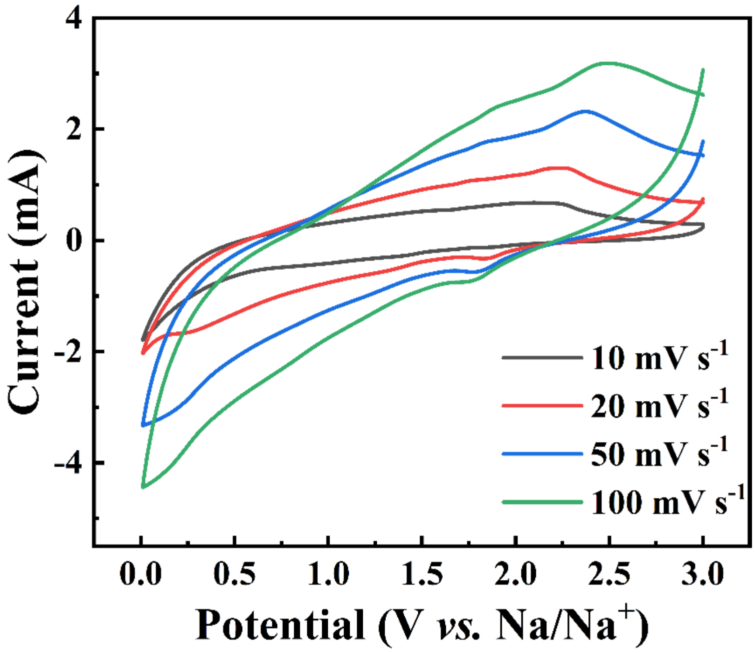

Figure S15. CV curves of E-1T MoS_2 electrode within the range of 10-100 mV s^{-1}.

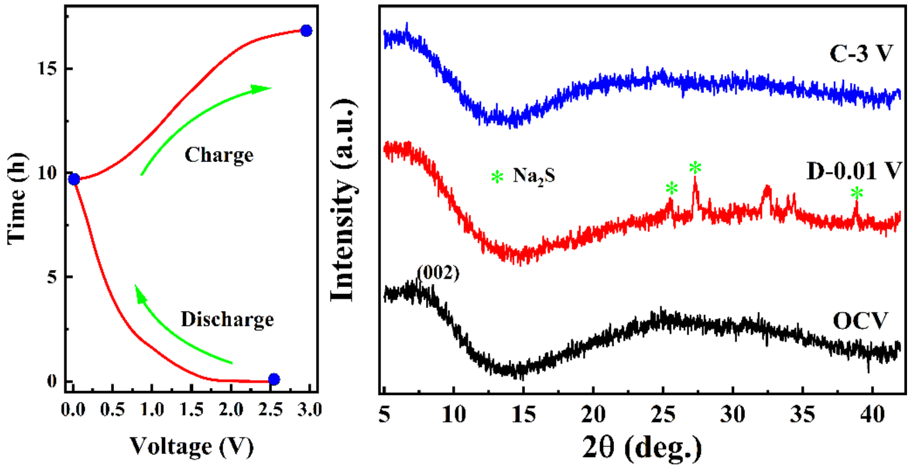

Figure S16. Ex-situ XRD patterns of E-1T MoS_2 electrode at the different discharge/charge states.

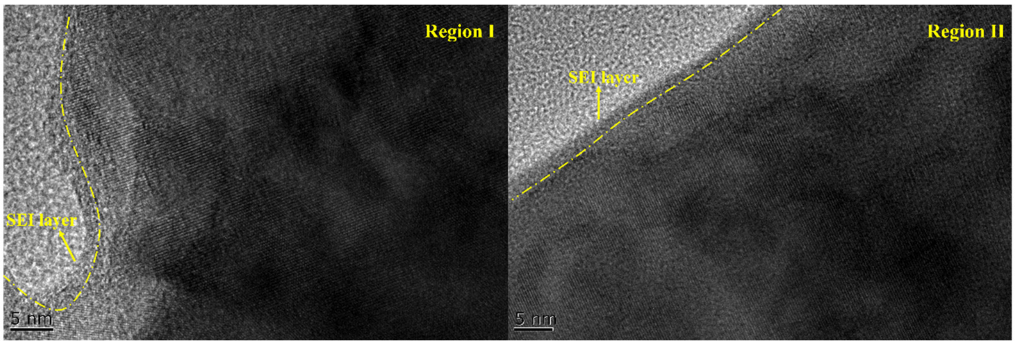

Figure S17. Ex-situ TEM images of the different regions in E-1T MoS_2 electrode at the fully discharged state.

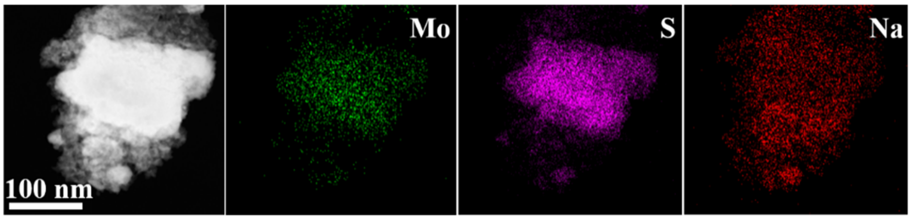

Figure S18. Ex-situ EDS mapping images of E-1T MoS_2 electrode at fully desodiation state.

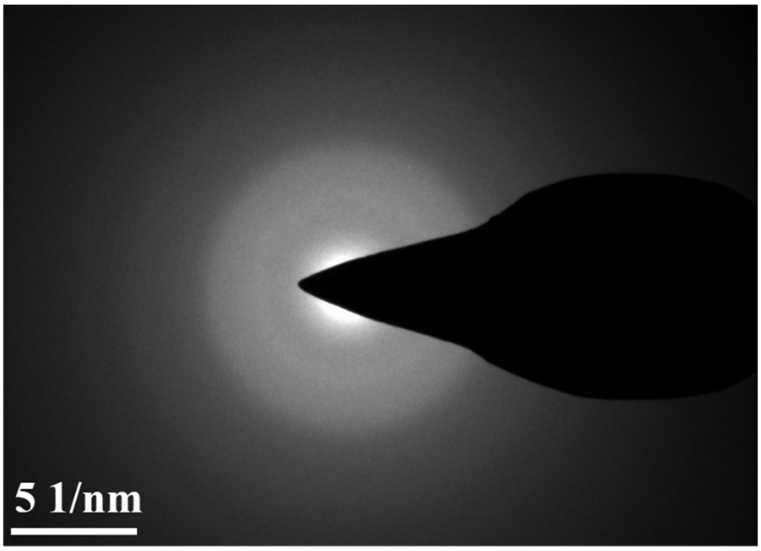

Figure S19. Ex-situ SAED pattern of E-1T MoS_2 electrode at fully desodiation state.

Table S1. Comparison of synthesis efficiency and 1T phase purity for E-1T MoS_2 and previously reported 1T-MoS_2 samples.

**Samples**	**Synthesis efficiency**	**1T phase purity**
E-1T MoS_2 (this work)	High (one-step)	70%
1T-P-MoS_2 (Ref. [33])	Medium (two-step)	54%
TiO-1TMoS_2 (Ref. [34])	Low (Muti-step)	100%

Note S1 The calculation process of loading mass of electrode material.

Assume that the quality of the total wafer electrode with the area of about 1 cm^2 is m_1, the quality of the corresponding Cu foil substrate is m_2, the quality of coating layer is m_3 and the quality of active material is m_4.

Thus, we can deduce $m_3 = m_1 – m_2$.

Based on the fact that the working electrode was fabricated by mixing the active materials, Super P, and sodium carboxymethyl cellulose (CMC) in a weight ratio of 8:1:1.

So, the loading mass of active material can be determined, $m_4 = m_3 * 0.8 = (m_1 - m_2) * 0.8$.

Note S2 The calculation process of d-spacings of the samples.

For the 2H MoS_2 sample, the 2θ of (002) peak is about 14.1°.

According to Bragg's law, $2d\sin\theta = \lambda$.

Because the wavelength λ (K_α) = 1.54154 Å.

So, we can deduce the corresponding $d \approx 0.63$ nm.

For the E-1T MoS_2 sample, the 2θ of (002) peak is about 8.8°.

Similarly, we can deduce the corresponding $d \approx 1.0$ nm.

Thus, we deduced that the obvious left shift of (002) peak could be originated from the intercalation of ethanol molecules.

Note S3 The calculation of coulombic efficiency.

Regarding to the anode material for SIBs, the coulombic efficiency (%) = charge specific capacity/discharge specific capacity *100%.

Note S4 The fitting method of pseudocapacitive contributions.

To further understand the origin of pseudocapacitive contributions, we provided the detailed pseudocapacitive fitting process as follows:

First, the fitting of pseudocapacitive contributions is based on the following equation as shown in the manuscript:

$i\ (V) = k_1 v + k_2 v^{1/2}$ (1)

where $i\ (V)$ stands for the current value at any voltage, $k_1 v$ represents pseudocapacitive contribution, and $k_2 v^{1/2}$ corresponds to diffusion-controlled contribution.

Then, equation (1) can be transformed into the following equation (2) by dividing both sides by $v^{1/2}$:

$i\ (V)/v^{1/2} = k_1 v^{1/2} + k_2$ (2)

Next, we can set $v^{1/2} = x$, and $i\ (V)/v^{1/2} = y$. Then, the equation (2) becomes the following equation (3):

$y = k_1 x + k_2$ (3)

Next, we need to transpose the original data presented in the Screenshot 1, then take the square root of the first column v to obtain $v^{1/2}$, and divide i in each of the other columns by the obtained $v^{1/2}$ in the first column to further achieve $i/v^{1/2}$ as shown in Screenshot 2.

Then, we perform the linear fitting of all data based on equation (3) to obtain a column of slope values, that is a set of k_1 values at each voltage as shown in Screenshot 3.

Finally, by multiplying k_1 by a certain scanning rate, we can obtain the pseudocapacitive contribution at the corresponding scanning rate as shown in the following equation (4):

Pseudocapacitive contribution(%) = Area(k_1v) / Area(i_v) $\times$100% (4)

where Area(k_1v) represents the absolute area enclosed by the CV curve formed by the k_1v current portion, and Area(i_v) stands for the absolute area enclosed by the CV curve at the scanning rate (v).

Thus, we can obtain the accurate pseudocapacitive contributions are 73.4%, 74.9%, 78.2%, 82.1%, 88.4% and 95.4% at the scanning rates of 0.2, 0.4, 0.6, 0.8, 1.0 and 1.2 mV s^{-1}, respectively.

Given the considerable computational load, domestic researchers have developed detailed calculation tutorial and software to facilitate the quick acquisition of pseudocapacitive fitting results. While, the working principle still relies on the aforementioned fitting method.

(Detailed calculation tutorials and calculation software see the following websites: https://mp.weixin.qq.com/s/GnOsPXmiCt66SegJGETOlg;http://www.upub.online/k1v2.6.html.)

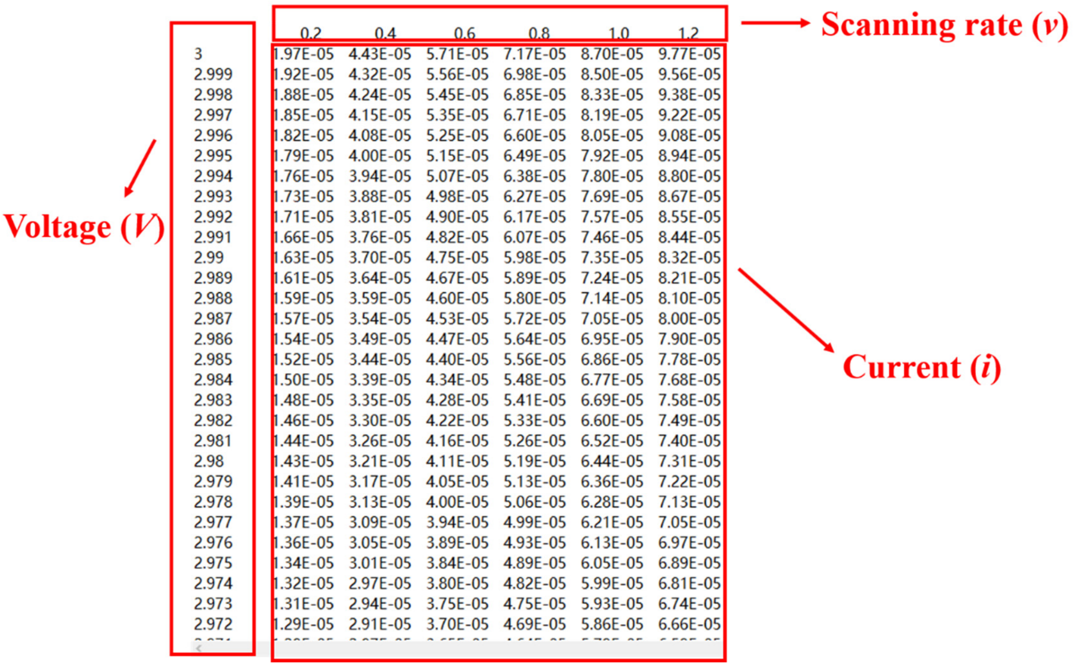

	0.2	0.4	0.6	0.8	1.0	1.2
3	1.97E-05	4.43E-05	5.71E-05	7.17E-05	8.70E-05	9.77E-05
2.999	1.92E-05	4.32E-05	5.56E-05	6.98E-05	8.50E-05	9.56E-05
2.998	1.88E-05	4.24E-05	5.45E-05	6.85E-05	8.33E-05	9.38E-05
2.997	1.85E-05	4.15E-05	5.35E-05	6.71E-05	8.19E-05	9.22E-05
2.996	1.82E-05	4.08E-05	5.25E-05	6.60E-05	8.05E-05	9.08E-05
2.995	1.79E-05	4.00E-05	5.15E-05	6.49E-05	7.92E-05	8.94E-05
2.994	1.76E-05	3.94E-05	5.07E-05	6.38E-05	7.80E-05	8.80E-05
2.993	1.73E-05	3.88E-05	4.98E-05	6.27E-05	7.69E-05	8.67E-05
2.992	1.71E-05	3.81E-05	4.90E-05	6.17E-05	7.57E-05	8.55E-05
2.991	1.66E-05	3.76E-05	4.82E-05	6.07E-05	7.46E-05	8.44E-05
2.99	1.63E-05	3.70E-05	4.75E-05	5.98E-05	7.35E-05	8.32E-05
2.989	1.61E-05	3.64E-05	4.67E-05	5.89E-05	7.24E-05	8.21E-05
2.988	1.59E-05	3.59E-05	4.60E-05	5.80E-05	7.14E-05	8.10E-05
2.987	1.57E-05	3.54E-05	4.53E-05	5.72E-05	7.05E-05	8.00E-05
2.986	1.54E-05	3.49E-05	4.47E-05	5.64E-05	6.95E-05	7.90E-05
2.985	1.52E-05	3.44E-05	4.40E-05	5.56E-05	6.86E-05	7.78E-05
2.984	1.50E-05	3.39E-05	4.34E-05	5.48E-05	6.77E-05	7.68E-05
2.983	1.48E-05	3.35E-05	4.28E-05	5.41E-05	6.69E-05	7.58E-05
2.982	1.46E-05	3.30E-05	4.22E-05	5.33E-05	6.60E-05	7.49E-05
2.981	1.44E-05	3.26E-05	4.16E-05	5.26E-05	6.52E-05	7.40E-05
2.98	1.43E-05	3.21E-05	4.11E-05	5.19E-05	6.44E-05	7.31E-05
2.979	1.41E-05	3.17E-05	4.05E-05	5.13E-05	6.36E-05	7.22E-05
2.978	1.39E-05	3.13E-05	4.00E-05	5.06E-05	6.28E-05	7.13E-05
2.977	1.37E-05	3.09E-05	3.94E-05	4.99E-05	6.21E-05	7.05E-05
2.976	1.36E-05	3.05E-05	3.89E-05	4.93E-05	6.13E-05	6.97E-05
2.975	1.34E-05	3.01E-05	3.84E-05	4.89E-05	6.05E-05	6.89E-05
2.974	1.32E-05	2.97E-05	3.80E-05	4.82E-05	5.99E-05	6.81E-05
2.973	1.31E-05	2.94E-05	3.75E-05	4.75E-05	5.93E-05	6.74E-05
2.972	1.29E-05	2.91E-05	3.70E-05	4.69E-05	5.86E-05	6.66E-05
[illegible]	[illegible]	[illegible]	[illegible]	[illegible]	[illegible]	[illegible]

Screenshot 1: partial original data of CV curves of E-1T MoS_2 electrode at 0.2-1.2 mV s^{-1} (Total original data amounts approach about 6000 rows, accompanying with voltage values from 3 to 0.01 V, and then back to 3 V, voltage interval: 0.001 V).

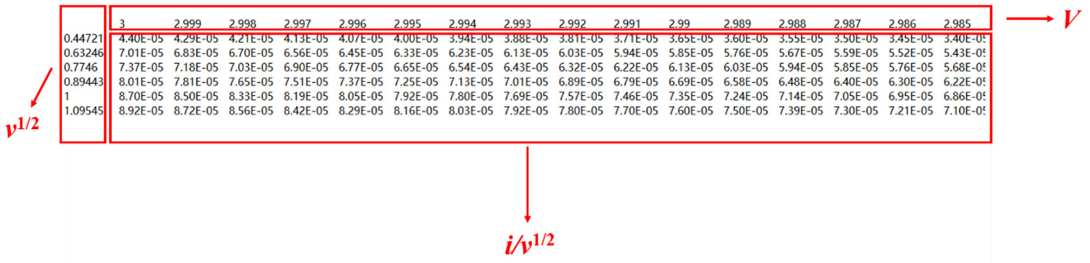

	3	2.999	2.998	2.997	2.996	2.995	2.994	2.993	2.992	2.991	2.99	2.989	2.988	2.987	2.986	2.985
0.44721	4.40E-05	4.29E-05	4.21E-05	4.13E-05	4.07E-05	4.00E-05	3.94E-05	3.88E-05	3.81E-05	3.71E-05	3.65E-05	3.60E-05	3.55E-05	3.50E-05	3.45E-05	3.40E-0
0.63246	7.01E-05	6.83E-05	6.70E-05	6.56E-05	6.45E-05	6.33E-05	6.23E-05	6.13E-05	6.03E-05	5.94E-05	5.85E-05	5.76E-05	5.67E-05	5.59E-05	5.52E-05	5.43E-0
0.7746	7.37E-05	7.18E-05	7.03E-05	6.90E-05	6.77E-05	6.65E-05	6.54E-05	6.43E-05	6.32E-05	6.22E-05	6.13E-05	6.03E-05	5.94E-05	5.85E-05	5.76E-05	5.68E-0
0.89443	8.01E-05	7.81E-05	7.65E-05	7.51E-05	7.37E-05	7.25E-05	7.13E-05	7.01E-05	6.89E-05	6.79E-05	6.69E-05	6.58E-05	6.48E-05	6.40E-05	6.30E-05	6.22E-0
1	8.70E-05	8.50E-05	8.33E-05	8.19E-05	8.05E-05	7.92E-05	7.80E-05	7.69E-05	7.57E-05	7.46E-05	7.35E-05	7.24E-05	7.14E-05	7.05E-05	6.95E-05	6.86E-0
1.09545	8.92E-05	8.72E-05	8.56E-05	8.42E-05	8.29E-05	8.16E-05	8.03E-05	7.92E-05	7.80E-05	7.70E-05	7.60E-05	7.50E-05	7.39E-05	7.30E-05	7.21E-05	7.10E-0

Screenshot 2: partial treated transposed data of CV curves for E-1T MoS_2 electrode at 0.2-1.2 mV s^{-1} (Total treated transposed data amounts approach about 6000 columns, accompanying with voltage values from 3 to 0.01 V, and then back to 3 V, voltage interval: 0.001 V).

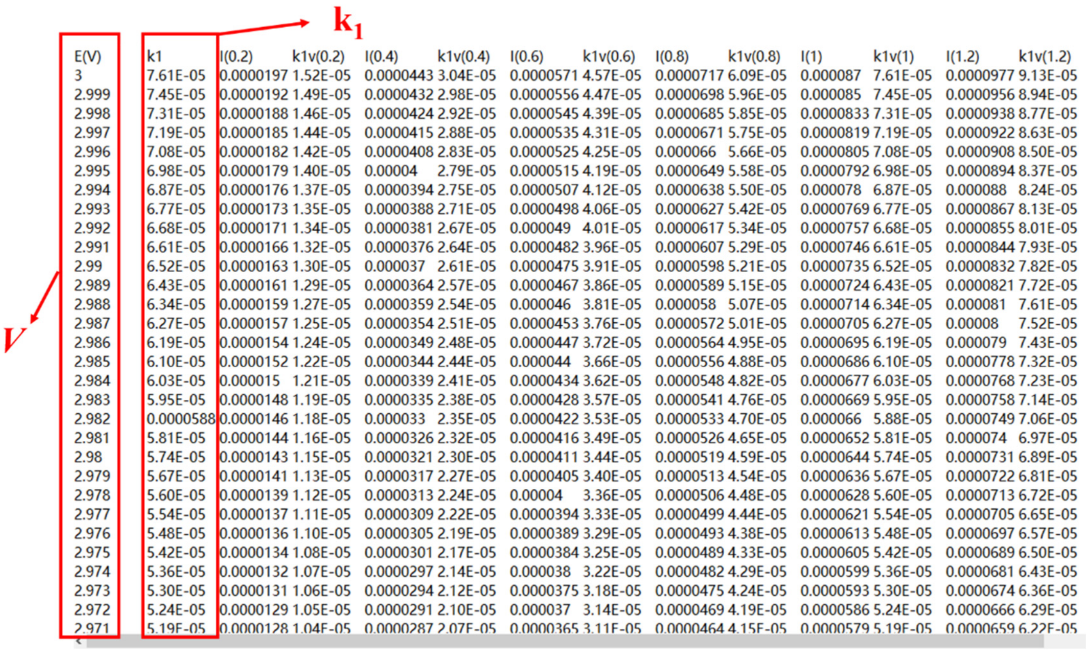

E(V)	k1	I(0.2)	k1v(0.2)	I(0.4)	k1v(0.4)	I(0.6)	k1v(0.6)	I(0.8)	k1v(0.8)	I(1)	k1v(1)	I(1.2)	k1v(1.2)
3	7.61E-05	0.0000197	1.52E-05	0.0000443	3.04E-05	0.0000571	4.57E-05	0.0000717	6.09E-05	0.000087	7.61E-05	0.0000977	9.13E-05
2.999	7.45E-05	0.0000192	1.49E-05	0.0000432	2.98E-05	0.0000556	4.47E-05	0.0000698	5.96E-05	0.000085	7.45E-05	0.0000956	8.94E-05
2.998	7.31E-05	0.0000188	1.46E-05	0.0000424	2.92E-05	0.0000545	4.39E-05	0.0000685	5.85E-05	0.0000833	7.31E-05	0.0000938	8.77E-05
2.997	7.19E-05	0.0000185	1.44E-05	0.0000415	2.88E-05	0.0000535	4.31E-05	0.0000671	5.75E-05	0.0000819	7.19E-05	0.0000922	8.63E-05
2.996	7.08E-05	0.0000182	1.42E-05	0.0000408	2.83E-05	0.0000525	4.25E-05	0.000066	5.66E-05	0.0000805	7.08E-05	0.0000908	8.50E-05
2.995	6.98E-05	0.0000179	1.40E-05	0.00004	2.79E-05	0.0000515	4.19E-05	0.0000649	5.58E-05	0.0000792	6.98E-05	0.0000894	8.37E-05
2.994	6.87E-05	0.0000176	1.37E-05	0.0000394	2.75E-05	0.0000507	4.12E-05	0.0000638	5.50E-05	0.000078	6.87E-05	0.000088	8.24E-05
2.993	6.77E-05	0.0000173	1.35E-05	0.0000388	2.71E-05	0.0000498	4.06E-05	0.0000627	5.42E-05	0.0000769	6.77E-05	0.0000867	8.13E-05
2.992	6.68E-05	0.0000171	1.34E-05	0.0000381	2.67E-05	0.000049	4.01E-05	0.0000617	5.34E-05	0.0000757	6.68E-05	0.0000855	8.01E-05
2.991	6.61E-05	0.0000166	1.32E-05	0.0000376	2.64E-05	0.0000482	3.96E-05	0.0000607	5.29E-05	0.0000746	6.61E-05	0.0000844	7.93E-05
2.99	6.52E-05	0.0000163	1.30E-05	0.000037	2.61E-05	0.0000475	3.91E-05	0.0000598	5.21E-05	0.0000735	6.52E-05	0.0000832	7.82E-05
2.989	6.43E-05	0.0000161	1.29E-05	0.0000364	2.57E-05	0.0000467	3.86E-05	0.0000589	5.15E-05	0.0000724	6.43E-05	0.0000821	7.72E-05
2.988	6.34E-05	0.0000159	1.27E-05	0.0000359	2.54E-05	0.000046	3.81E-05	0.000058	5.07E-05	0.0000714	6.34E-05	0.000081	7.61E-05
2.987	6.27E-05	0.0000157	1.25E-05	0.0000354	2.51E-05	0.0000453	3.76E-05	0.0000572	5.01E-05	0.0000705	6.27E-05	0.00008	7.52E-05
2.986	6.19E-05	0.0000154	1.24E-05	0.0000349	2.48E-05	0.0000447	3.72E-05	0.0000564	4.95E-05	0.0000695	6.19E-05	0.000079	7.43E-05
2.985	6.10E-05	0.0000152	1.22E-05	0.0000344	2.44E-05	0.000044	3.66E-05	0.0000556	4.88E-05	0.0000686	6.10E-05	0.0000778	7.32E-05
2.984	6.03E-05	0.000015	1.21E-05	0.0000339	2.41E-05	0.0000434	3.62E-05	0.0000548	4.82E-05	0.0000677	6.03E-05	0.0000768	7.23E-05
2.983	5.95E-05	0.0000148	1.19E-05	0.0000335	2.38E-05	0.0000428	3.57E-05	0.0000541	4.76E-05	0.0000669	5.95E-05	0.0000758	7.14E-05
2.982	0.0000588	0.0000146	1.18E-05	0.000033	2.35E-05	0.0000422	3.53E-05	0.0000533	4.70E-05	0.000066	5.88E-05	0.0000749	7.06E-05
2.981	5.81E-05	0.0000144	1.16E-05	0.0000326	2.32E-05	0.0000416	3.49E-05	0.0000526	4.65E-05	0.0000652	5.81E-05	0.000074	6.97E-05
2.98	5.74E-05	0.0000143	1.15E-05	0.0000321	2.30E-05	0.0000411	3.44E-05	0.0000519	4.59E-05	0.0000644	5.74E-05	0.0000731	6.89E-05
2.979	5.67E-05	0.0000141	1.13E-05	0.0000317	2.27E-05	0.0000405	3.40E-05	0.0000513	4.54E-05	0.0000636	5.67E-05	0.0000722	6.81E-05
2.978	5.60E-05	0.0000139	1.12E-05	0.0000313	2.24E-05	0.00004	3.36E-05	0.0000506	4.48E-05	0.0000628	5.60E-05	0.0000713	6.72E-05
2.977	5.54E-05	0.0000137	1.11E-05	0.0000309	2.22E-05	0.0000394	3.33E-05	0.0000499	4.44E-05	0.0000621	5.54E-05	0.0000705	6.65E-05
2.976	5.48E-05	0.0000136	1.10E-05	0.0000305	2.19E-05	0.0000389	3.29E-05	0.0000493	4.38E-05	0.0000613	5.48E-05	0.0000697	6.57E-05
2.975	5.42E-05	0.0000134	1.08E-05	0.0000301	2.17E-05	0.0000384	3.25E-05	0.0000489	4.33E-05	0.0000605	5.42E-05	0.0000689	6.50E-05
2.974	5.36E-05	0.0000132	1.07E-05	0.0000297	2.14E-05	0.000038	3.22E-05	0.0000482	4.29E-05	0.0000599	5.36E-05	0.0000681	6.43E-05
2.973	5.30E-05	0.0000131	1.06E-05	0.0000294	2.12E-05	0.0000375	3.18E-05	0.0000475	4.24E-05	0.0000593	5.30E-05	0.0000674	6.36E-05
2.972	5.24E-05	0.0000129	1.05E-05	0.0000291	2.10E-05	0.000037	3.14E-05	0.0000469	4.19E-05	0.0000586	5.24E-05	0.0000666	6.29E-05
2.971	5.19E-05	0.0000128	1.04E-05	0.0000287	2.07E-05	0.0000365	3.11E-05	0.0000464	4.15E-05	0.0000579	5.19E-05	0.0000659	6.22E-05

Screenshot 3: partial k_1 fitting results of CV curves for E-1T MoS_2 electrode at 0.2-1.2 mV s^{-1} (Total k_1 fitting data amounts approach about 6000 rows, accompanying with voltage values from 3 to 0.01 V, and then back to 3 V, voltage interval: 0.001 V).
